# Application of insulin-like growth factor-1 in the treatment of inner ear disorders

**DOI:** 10.3389/fphar.2014.00208

**Published:** 2014-09-10

**Authors:** Norio Yamamoto, Takayuki Nakagawa, Juichi Ito

**Affiliations:** Department of Otolaryngology, Head and Neck Surgery, Graduate School of Medicine, Kyoto UniversityKyoto Japan

**Keywords:** apoptosis, cell cycle, clinical trial, MEK/ERK pathway, Netrin1, PI3K/Akt pathway, sensorineural hearing loss

## Abstract

Sensorineural hearing loss (SNHL) is considered an intractable disease, given that hair and supporting cells (HCs and SCs) of the postnatal mammalian cochlea are unable to regenerate. However, with progress in regenerative medicine in the 21st century, several innovative approaches for achieving regeneration of inner ear HCs and SCs have become available. These methods include stem cell transplantation, overexpression of specific genes, and treatment with growth factors. Insulin-like growth factor-1 (IGF-1) is one of the growth factors that are involved in the development of the inner ear. Treatment with IGF-1 maintains HC numbers in the postnatal mammalian cochlea after various types of HC injuries, with activation of two major pathways downstream of IGF-1 signaling. In the aminoglycoside-treated neonatal mouse cochlear explant culture, promotion of the cell-cycle in SCs as well as inhibition of HC apoptosis was observed in the IGF-1-treated group. Activation of downstream molecules was observed in SCs and, in turn, SCs contribute to the maintenance of HC numbers. Using comprehensive analysis of the gene expression, the candidate effector molecules of the IGF-1 signaling pathway in the protection of HCs were identified as Netrin1 and Gap43. Based on these studies, a clinical trial has sought to investigate the effects of IGF-1 on SNHL. Sudden SNHL (SSHL) that was refractory to systemic steroids was treated with IGF-1 in a gelatin hydrogel and the outcome was compared with a historical control of hyperbaric oxygen therapy. The proportion of patients showing hearing improvement was significantly higher in the IGF-1-treatment group at 24 weeks after treatment than in the control group. A randomized clinical trial is ongoing to compare the effect of IGF-1 treatment with that of intra-tympanic steroids for SSHL that is refractory to systemic steroids.

## INTRODUCTION

Sensorineural hearing loss (SNHL) is a common disability. It includes age-related hearing loss, noise-induced hearing loss, genetic hearing loss, sudden sensorineural hearing loss (SSHL), and hearing loss caused by ototoxic drugs (for example, aminoglycosides). In the United States, about 63% of people older than 70 years have hearing loss (Lin et al., 2011). Profound, early onset deafness is found in 4–11 per 10,000 children and in 50% of such cases, the causes are genetic (Marazita et al., 1993). SSHL occurs in 5–20 cases per 100,000 persons annually (Byl, 1984). Despite the high prevalence, no effective treatment has been established for SNHL, except for cochlear implants in profound SNHL or steroid treatment in the cases of acute hearing loss, such as SSHL. Most SNHL cases are caused by the loss or functional impairment of hair cells (HCs) in the cochlea, but mammalian HCs have not been considered to be capable of proliferation during postnatal stage (Ruben, 1967). Consequently, HCs never regenerate in the cochlea, a hearing organ in the inner ear, although HCs in the vestibular organs (utricle and saccule) in the inner ear have minimal capacity for regeneration (Forge et al., 1993; Warchol et al., 1993). This fact has prevented development of effective methods for treating SNHL. In contrast to mammals, avian inner ear HCs have been shown to be able to regenerate even after birth (Corwin and Cotanche, 1988; Jorgensen and Mathiesen, 1988; Ryals and Rubel, 1988). Regeneration of avian HCs is based on both proliferation (Corwin and Cotanche, 1988; Ryals and Rubel, 1988) and transdifferentiation (Adler and Raphael, 1996; Baird et al., 1996) of supporting cells (SCs) that surround HCs. Thus, SCs are considered to be a source of HC regeneration.

As regenerative medicine emerged in the 21st century, many researchers have attempted to regenerate mammalian HCs using various strategies: stem cell transplantation, overexpression of specific genes, and treatment with growth factors. Tissue-engineering technology, which is one of the most powerful tools of regenerative medicine, is based on three important factors, namely, isolated cells, tissue-inducing substances, and scaffold or matrices into which cells are placed (Langer and Vacanti, 1993), all of which are also necessary for regenerative medicine.

Tissue-inducing substances also include growth factors, which are humoral factors that bind to their specific receptors on the cell membrane to activate intracellular signaling in order to exert their effects. Since growth factors were first extracted from exocrine or endocrine organs in the 1960’s and 1970’s (Cohen, 1962; Armelin, 1973), they have been demonstrated to regulate survival, proliferation, and differentiation of various types of cells in various organs. These functions prompted the application of some growth factors to regenerative medicine. For instance, basic fibroblast growth factor enhances proliferation of epidermal cells, inspiring its use in the treatment of pressure ulcers and other types of skin ulcer. From the study of hematopoietic stem cells and various precursor cells of the hematopoietic system, various growth factors have been identified as regulating the induction and proliferation of specific lineages of hematopoietic cells. Among these growth factors, erythropoietin and granulocyte-colony stimulating factor are clinically used to treat anemia and neutropenia, respectively. These are good examples of the application of growth factors in regenerative medicine.

In this article, after first briefly reviewing the role of growth factors, particularly insulin-like growth factor-1 (IGF-1), in the inner ear, we summarize the application of IGF-1 to the treatment of inner ear disorders in animal models, the mechanisms underlying the effects of IGF-1 on the inner ear, and a clinical trial of IGF-1 in human SSHL cases.

## GROWTH FACTORS AND THE INNER EAR

It has been investigated whether growth factors could induce proliferation of HCs, which have been reported to lose their proliferative ability from the embryonic stage (Ruben, 1967). Growth factors were expected to induce HC proliferation during the postnatal stage because they have a marked capacity for inducing proliferation in treated cells. When neonatal rat utricle cells were dissociated and cultured, the effect of various types of growth factors on the proliferation of utricular cells was investigated quantitatively (Zheng et al., 1997). Among several growth factors tested, epidermal growth factor, transforming growth factor α, basic fibroblast growth factor, and IGF-1 were able to induce proliferation. In particular, combination of basic fibroblast growth factor and IGF-1 or transforming growth factors α had additive effects. Similar mitogenic effects were confirmed for epidermal growth factor and transforming growth factor α in vestibular organ culture (Lambert, 1994; Yamashita and Oesterle, 1995). Moreover, epidermal growth factor caused supernumerary HC formation even in neonatal mouse cochlear organ culture (Lefebvre et al., 2000).

## INSULIN-LIKE GROWTH FACTOR-1 AND ITS DOWNSTREAM SIGNALING

Insulin-like growth factor-1 was originally found in human serum as a factor that stimulates the incorporation of sulfate into cartilage (Salmon and Daughaday, 1957). Later, it was shown that its insulin-like activity was not suppressed by anti-insulin antibodies (Froesch et al., 1963) although its structure was similar to that of proinsulin (Rinderknecht and Humbel, 1976a). The insulin-like activity of IGF-1 was 60 times lower than that of insulin but, in contrast, the [^3^H]-thymidine incorporation activity (proliferation-induction activity) of IGF-1 was 50 times higher than that of insulin (Rinderknecht and Humbel, 1976b). IGF-1 is a small peptide, comprising 70 amino acids (Rinderknecht and Humbel, 1978), which acts as an anabolic and mitogenic effector of growth hormone (Laron, 1999). IGF-1 in the serum is synthesized mainly in the liver and most of the circulating IGF-1 forms a complex with its binding proteins, IGFBP1–6 (Jones and Clemmons, 1995) that modify the activity of IGF-1.

**FIGURE 1:**
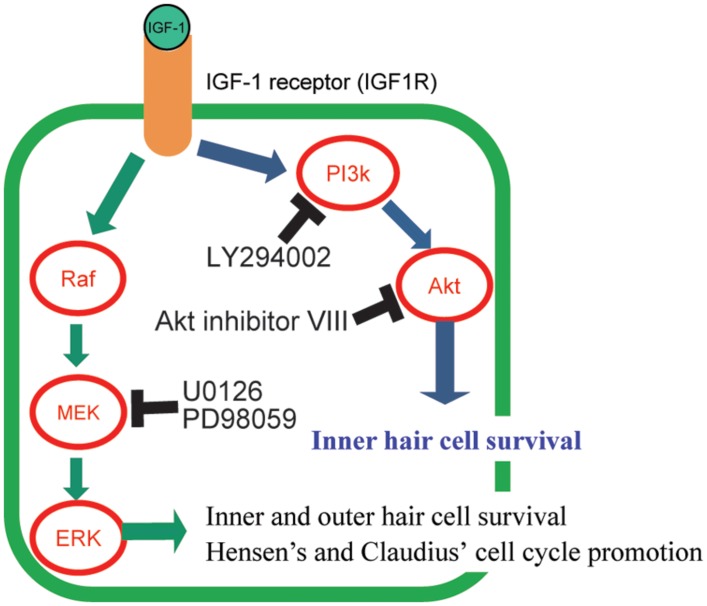
**Graphical summary of the signaling pathways of IGF-1 and its downstream effectors, and its effect on the protection of hair cells (HC_**S**_).** The mechanisms of HC number maintenance by IGF-1 were elucidated using several specific inhibitors (LY294002, Akt inhibitor VIII, U0126, and PD98059) of the downstream molecules in IGF-1 signaling pathways. IGF-1 protects HC_S_ against aminoglycosides through activation of both the MEK/ERK and PI3K/AKT pathways. Akt is involved in the survival of only inner HC_S_. MEK/ERK causes cell cycle promotion in Hensen’s and Claudius’ cells.

Insulin-like growth factor-1 exerts its action through its receptor, IGF1 receptor (IGF1R). IGF1R belongs to a family of tyrosine kinase receptors. Once IGF-1 binds to IGF1R, it induces autophosphorylation on the intracellular tyrosine residues (Kato et al., 1994; **Figure 1**). Activated IGF1R interacts with and phosphorylates other intracellular adaptor molecules, viz., the insulin receptor substrate (IRS) 1–4 and Src homology collagen (Shc) protein (Myers et al., 1993; Blakesley et al., 1996; White, 1997). Phosphorylated IRSs activate phosphatidylinositol-3 kinase (PI3K) and lead to the formation of membrane-associated phosphorylated inositols. These molecules activate phosphoinositide-dependent kinase, which phosphorylates several other protein kinases, such as Akt (PI3K/Akt pathway; **Figure 1**) and protein kinase C (Vanhaesebroeck and Alessi, 2000). PI3K activation is involved in the inhibition of apoptosis, regulation of gene transcription, and mitogenesis (Shepherd et al., 1998). IGF1R activates another pathway through the recruitment to both IRS-1 and Shc of Sos via the SH2 domain of Grb2 (Yamauchi and Pessin, 1994). This recruitment leads to the activation of the small G-protein, Ras, which in turn causes the activation of Raf. Raf phosphorylates MAPK/ERK kinase (MEK) and MEK activates the extracellular-signal-regulated kinase (ERK) in the MEK/ERK pathway (**Figure 1**; Laviola et al., 2007). This pathway is mainly involved in mitogenic (Pagès et al., 1993), transcriptional (Laviola et al., 2007), or anti-apoptotic responses (Holmström et al., 1999; Finlay et al., 2000).

As an effector of growth hormone, IGF-1 is involved in the anabolic growth of various organs. In addition to this original role, IGF-1 also regulates, for example in the central nervous system, neuronal survival, neurogenesis, angiogenesis, excitatory, and inhibitory neurotransmission, regulation of food intake, and cognition (Werner and Leroith, 2014). Since recombinant IGF-1 has been synthesized, it has been used clinically in replacement therapy in patients with primary growth hormone-resistance (Laron syndrome) where IGF-1 is not synthesized (Laron, 1999). However, recombinant IGF-1 has been evaluated clinically for the treatment of other diseases. It has been reported to reduce the requirement for insulin in type I diabetes mellitus, to reduce the serum levels of lipoproteins, to increase the glomerular filtration and the tubular reabsorption of phosphate, and to slow the progression of amyotrophic lateral sclerosis (Laron, 1999). However, because the mitogenic effects of IGF-1 are involved in the growth regulation of breast (Wolf et al., 1997) and prostate cancer (Chan et al., 1998; Wang and Wong, 1998) and because patients with IGF-1 hypersecretion (acromegaly) have a higher incidence of large bowel polyposis, the use of recombinant IGF-1 should be avoided in such patients or in those with a family history of these tumors.

## IGF-1 AND THE INNER EAR

Since Leon et al. (1995) reported that IGF-1 promotes the growth of chicken otocysts by inducing cell proliferation during the early developmental stage of the inner ear, many studies have shown that IGF-1 and its signaling system plays important roles in the development or maintenance of the inner ear, and, as a result, in hearing. In human beings, SNHL occurs in patients with mutations in *Igf1* gene (Woods et al., 1996; Bonapace et al., 2003; Walenkamp et al., 2005), primary IGF-1 deficiency (Attias et al., 2012), or low serum IGF-1 levels due to other genetic defects (Barrenas et al., 2000; Johnson et al., 2007), indicating the importance of IGF-1 in hearing. Replacement therapy using recombinant IGF-1 rescues patients from the hearing loss in Laron syndrome (Attias et al., 2012). The SNHL in Laron syndrome patients is attributed to cochlear dysfunction, based on otoacoustic emission test results (Attias et al., 2012). However, in a mouse model of IGF-1 deficiency, that is in IGF-1 knockout mice (Camarero et al., 2001, 2002; Cediel et al., 2006; Riquelme et al., 2010), the causes of SNHL were reported as the loss of spiral ganglion neurons, abnormal myelination of the cochlear nerve, and degeneration of the stria vascularis, based on the results of morphological (Camarero et al., 2001, 2002) and auditory brain stem response studies (Cediel et al., 2006; Riquelme et al., 2010). The stria degeneration becomes apparent from the age of 3 months in IGF-1 knockout mice although the loss of spiral ganglion cells commences from the age of 3 weeks (Camarero et al., 2002; Riquelme et al., 2010). Knocking out of *Irs2*, a gene encoding an intracellular adaptor molecule of IGF1R, also showed similar inner ear phenotypes to IGF-1 knockout mice (Murillo-Cuesta et al., 2012). In contrast to IGF-1 knockout mice, deletion of *Igfr1* caused inner ear anomalies (short cochlear duct, truncated lateral semicircular canal, and hypomorphic posterior semicircular canal), the delay of maturation of HCs and SCs in the cochlea, and reduced proliferation of prosensory cells in the development of the inner ear (Okano et al., 2011). Unfortunately, hearing phenotypes and mature inner ear morphology could not be studied because of the embryonic lethality caused by *Igf1r* knockout. Comparable phenotypes were reproduced with the treatment of IGF1R inhibitors on the embryonic cochlear explant culture. These IGF1R-mediated effects were observed in the middle to late stage of cochlear development and were dependent on the PI3K/Akt pathway but not on the MEK/ERK pathway, as indicated by detection of phosphorylated downstream of IGF1R and by inhibitor experiments (Okano et al., 2011). In contrast, the growth and proliferative effects of IGF-1 on otocysts and its survival effects in proliferative otic neuroblasts during the early developmental stage were dependent on the MEK/ERK pathway (Leon et al., 1998; Sanz et al., 1999; Magarinos et al., 2010) and the PI3K/AKT pathway (Aburto et al., 2012), respectively. The effector molecules of IGF-1 in the late stage of cochlear development have been studied using comprehensive gene expression analysis; several transcriptional factors (FoxM1, Mef2a, and Mef2d) have been identified as effectors of IGF-1 signaling (Sanchez-Calderon et al., 2010).

The physiological functions of IGF-1 and its downstream signaling included the induction of proliferation in the development of the inner ear. Since postnatal inner ear HCs or SCs lose their proliferation potency, particularly in the cochlea (Ruben, 1967), and as that prevents the regeneration of mammalian HCs after birth, IGF-1 may contribute to postnatal HC regeneration in mammals. Additionally, survival effects (anti-apoptotic effects) may contribute to the prevention of HC death after exposure to several types of conditions that cause inner ear pathology, including noise, ischemia, and toxic drug treatment, because these conditions usually cause apoptosis in HCs. Addition of IGF-1 to explant cultures of mature avian vestibular epithelia caused three-fold upregulation of DNA synthesis after a 48-h treatment but the numbers of [^3^H]-thymidine-incorporating cells were similar between 5-h- and 2-day-treatments, suggesting that IGF-1 did not contribute to the survival of the sensory epithelium (Oesterle et al., 1997). Explant cultures of avian auditory epithelia did not show this increase in DNA synthesis (Oesterle et al., 1997). Induction of proliferation was observed in neonatal rat dissociated vestibular SCs that had been treated with IGF-1 although other growth factors, including epidermal growth factor and transforming growth factor α, did not show any proliferative effects (Zheng et al., 1997). This experiment suggested that IGF-1 was a candidate molecule for inducing regeneration of even postnatal mammalian inner ear HCs.

Explant culture of adult mammalian cochlea causes loss of outer HCs due to apoptosis even after a short culture period. Treatment with IGF-1 as well as acidic fibroblast growth factor, epidermal growth factor, and transforming growth factors β1 prevented the loss of outer HCs, suggesting the survival effect of IGF-1 on mature mammalian outer HCs (Malgrange et al., 2002). The survival effect of IGF-1 against aminoglycoside-induced severe HC loss was observed in explant cultures of postnatal mammalian utricle (Park et al., 2007; Angunsri et al., 2011) and *in vivo* vestibular systems of mature guinea pigs (Kopke et al., 2001). In the case of the *in vivo* experiments, the perilymph of the mature guinea pig inner ear was infused with mixture of transforming growth factor α, IGF-1, and retinoic acid after injury induced by aminoglycoside, and functional as well as morphological recovery of HCs was observed (Kopke et al., 2001).

## *IN VIVO* APPLICATION OF IGF-1 FOR COCHLEAR DAMAGE

The successful application of IGF-1 to the treatment of aminoglycoside-induced mammalian vestibular HC damage suggested that IGF-1 was an effective molecule for use in inner ear disorders. However, unilateral peripheral vestibular damage can be compensated by the central nervous system and the demand for treatment of an affected vestibular system is less than in the case of the auditory system, where compensation never occurs. Moreover, unlike in the cochlea, mature mammalian vestibular sensory epithelia have the capacity to regenerate (Forge et al., 1993; Warchol et al., 1993) although this capacity is not markedly strong. Treatment of cochlear damage is more challenging. In a study of vestibular systems, IGF-1 was delivered as single recombinant molecule, because IGF-1 is a relatively stable molecule compared with insulin. However, the half-life of IGF-1 is about 8–16 h (Laron, 1999). For inner ear treatment, local application of IGF-1 is a more preferable drug delivery method compared with systemic delivery, because blood flow of inner ears is only 1/10000 to 1/1000000th of the systemic flow. Considering these conditions, gelatin hydrogel (Young et al., 2005) has been used to achieve efficient local application of IGF-1 into the animal or human cochlea (Iwai et al., 2006; Lee et al., 2007; Fujiwara et al., 2008; Nakagawa et al., 2010). In a gelatin hydrogel drug delivery system, sufficient IGF-1 levels are detected in the perilymph until at least 3 days after application of IGF-1 within the hydrogel to the round window of the cochleae (Lee et al., 2007).

First of all, IGF-1 was applied 3 days prior to noise exposure in mature rat (Iwai et al., 2006). Threshold shifts for 16 and 32 kHz were significantly smaller in the IGF-1 group at 7 days after noise exposure. Histological evaluation showed that outer HC loss was significantly milder in all turns of the cochleae in the IGF-1 group than in the control group. These results indicated that local application of IGF-1 had positive effects on the protection of outer HCs against noise exposure and, in turn, caused reduced threshold shifts in the hearing ability of adult rats. Next, IGF-1 was applied into the adult guinea pig cochlea 5 h after noise exposure (Lee et al., 2007). It is important to confirm whether the treatment had effects even after noise exposure because prophylactic application of a drug is sometimes difficult in clinical practice. Similar to the previous study, threshold shifts due to noise exposure were significantly attenuated and outer HC cell loss was also significantly reduced by the IGF-1 treatment. These two experiments clearly showed that IGF-1 application using gelatin hydrogel was a promising candidate treatment for noise-induced hearing loss.

Insulin-like growth factor-1 application has also been studied in a cochlear ischemia and reperfusion injury model in Mongolian gerbils (Fujiwara et al., 2008), which represents hearing loss similar to sudden deafness in humans (Hakuba et al., 1997; Koga et al., 2003). In this study, threshold shifts, assessed by auditory brain stem response, were significantly attenuated in an IGF-1-treatment group, similar to that observed in the noise exposure model. However, there were some points of difference between the effects of IGF-1 on noise exposure and ischemic injuries of the cochlea. First, threshold attenuation occurred 1 day after the injury in the ischemic and reperfusion model. In contrast, it occurred only 14 days after noise exposure. Second, rescued HC types were different between these two conditions: inner and outer HC loss was attenuated in ischemic and noise exposure models, respectively. These results suggested several points about the effect of IGF-1 on SNHL. (1) IGF-1 has the ability to affect both the early and late phase of SNHL. (2) IGF-1 treatment has potency to protect both inner and outer HCs from damage. (3) IGF-1 has effects on different mammals, viz., rats, guinea pigs, and Mongolian gerbils.

## MECHANISMS BY WHICH HC NUMBERS ARE MAINTAINED BY IGF-1 TREATMENT

Cochlear explant culture systems are suitable for analyzing the mechanisms underlying treatment effects because timing of drug application or sample collection is easier to control than *in vivo* experimental models. Therefore, the mechanisms underlying the positive effects of IGF-1 on cochlear HCs were evaluated using cochlear explant cultures (Hayashi et al., 2013). In this system, cochlear explant cultures were established from neonatal mice and aminoglycoside was used to induce damage to HCs. IGF-1 treatment resulted in the maintenance of both inner and outer HC numbers after aminoglycoside treatment, which was similar to its effects on noise exposure and ischemic injury in *in vivo* experiments. Specific inhibitors of PI3K, Akt, or ERK were used with IGF-1 to elucidate which downstream signaling pathways of IGF1R were involved in the protective effects of IGF-1 (**Figure 1**). Different agents were used to inhibit either the PI3K/Akt or MEK/ERK pathways. The PI3K inhibitor, LY294002, and the Akt inhibitor, Akt inhibitor VIII, were used to inhibit the PI3K/Akt pathway. For the MEK/ERK pathway, two different ERK inhibitors (U0126 and PD98059) were used. Either treatment caused attenuation of HC-protection effects, indicating that both pathways were involved in the maintenance of HC numbers. These results are different from the roles of IGF-1 in the development the cochlea, where only the PI3K/Akt pathway was involved at a later stage (Okano et al., 2011), and suggested that treatment with IGF-1 is a more efficient method for protecting HCs than the activation of a single signal cascade although the activation of a single pathway is effective for HC protection (Battaglia et al., 2003; Jiang et al., 2006; Selivanova et al., 2007). Among several inhibitors used, Akt inhibitor treatment attenuated only the inner HC protection effect, suggesting that Akt is involved in only the maintenance of inner HCs. A study of the localization of phosphorylated Akt and phosphorylated ERK using antibodies to these molecules determined that both Akt and ERK were activated not in HCs, but in SCs. Akt was activated in the inner sulcus cells that are located adjacent to inner HCs. In contrast, ERK was activated in Hensen’s and Claudius’ cells that are located lateral to the outer HCs. The localization of phosphorylated Akt-positive cells was consistent with the experiment in which an Akt inhibitor was used and indicated that Akt is an important signal for the protection of inner HCs. These results also raised the question about how IGF-1 protected HCs. One of the hypotheses is that IGF-1 acts on SCs and SCs, in turn, secrete some factors or express signal-transducing proteins on the membrane and these factors or proteins induce HC maintenance or regeneration.

There are two possible mechanisms involved in the maintenance of HC numbers. One is the inhibition of cell death and the other is the proliferation of HCs themselves or transdifferentiation of SCs into HCs after SC proliferation. Involvement of these two mechanisms has also been studied using cochlear explant cultures (Hayashi et al., 2013). These results showed that apoptosis of HCs was significantly inhibited in the IGF-1-treated group and, surprisingly, induction of cell-cycle promotion was also observed in the epithelium of postnatal cochlear explants. The latter result was inconsistent with the physiological characteristics of postnatal mammalian cochleae, but cell cycle promotion was confirmed by two different methods, i.e., 5-bromo-2′-deoxyuridine (BrdU) incorporation and phosphohistone H3 immunostaining. In addition, treatment with two different proliferation inhibitors, aphidicolin or L-mimosine, caused attenuation of only outer HC number maintenance. Together with the finding that all BrdU-positive cells were observed in Hensen’s and Claudius’ cells adjacent to outer HCs, this indicated that promotion of the cell cycle in the cochlea by IGF-1 contributed to maintenance of the outer HCs only and that transdifferentiation of SCs into HCs after SC proliferation may be the mechanism involved. The MEK/ERK pathway is likely to be involved in the cell cycle promotion in Hensen’s and Claudius’ cells, as deduced from two findings: phosphorylated ERK, which indicates the activation of the MEK/ERK pathway, was detected in Hensen’s and Claudius’ cells where BrdU incorporation and phophohistone H3 were detected, and the proliferation of SCs was caused by activation of the MEK/ERK pathway in chicken basilar papillae, resulting in the regeneration of HCs (Bell and Oberholtzer, 2010).

The target molecules of by which IGF-1 maintains the numbers of HCs were identified using comprehensive analysis of gene expression in explant cultures treated with IGF-1 (Hayashi et al., 2014). The expression of two molecules, Netrin1 and Gap43, was significantly up-regulated in the IGF-1-treated group and the result was confirmed by quantitative reverse transcriptase-polymerase chain reaction. Upregulation of gene expression was reduced by inhibition of both the MEK/ERK and PI3K/AKT pathways, indicating that the target molecules of IGF-1 were regulated by both pathways. Netrin1 is a secreted molecule that exerts its effects through its specific receptors. Originally, it was described as being involved in axon guidance of the nervous system (Hedgecock et al., 1990), but recent reports have shown its beneficial effects in injured organs, including the kidney. In the kidney, translation of Netrin1 is induced in response to reperfusion injury through the activation of the MEK/ERK pathway (Jayakumar et al., 2011), and Netrin1 increases the proliferation and migration of renal proximal tubular epithelial cells (Wang et al., 2009b). Moreover, overexpression of Netrin1 protects the kidney from ischemia and reperfusion injury (Wang et al., 2009a). Since the effect of HC protection by IGF-1 was achieved through cell cycle promotion and inhibition of apoptosis (Hayashi et al., 2013), Netrin1 is the probable effector molecule of IGF-1 during cochlear HC protection.

## CLINICAL TRIAL OF IGF-1 FOR SSHL

Based on these *in vivo* and *in vitro* studies, a clinical trial was performed to study the efficacy of IGF-1 in the treatment of SNHL (University Hospital Medical Information Network Clinical Trials Registry under trial registration number UMIN000000936; Nakagawa et al., 2010, 2012). In this clinical trial, 25 patients with SSHL that was refractory to systemic steroid therapy were recruited and IGF-1 was applied onto the round window membrane, using gelatin hydrogel, as performed in *in vivo* animal studies (Iwai et al., 2006; Lee et al., 2007; Fujiwara et al., 2008). The outcome of IGF-1 treatment was compared with that of a historical control after hyperbaric oxygen therapy where 66 out of 199 patients (33%) showed the hearing threshold improvement (Nakagawa et al., 2010). In this trial, improvement of hearing threshold was defined as recovery of more than 10 dB in the mean hearing level at the five frequencies tested (0.25, 0.5, 1.0, 2.0, and 4.0 kHz). At 12 and 24 weeks after IGF-1 treatment, the proportions of patients showing hearing improvement was 48 and 56%, respectively. No serious adverse effects were observed. The proportion of hearing improvement at 24 weeks after IGF-1 treatment was significantly better than that of the historical control. Analyses of recovery of threshold in each frequency tested revealed that the thresholds for lower frequencies recovered better than those for higher frequencies (Nakagawa et al., 2012). Average recovery in pure tone audiometry thresholds over the five frequencies tested was 11.9 dB (Nakagawa et al., 2012), which was comparable to the average threshold recovery previously reported for intra-tympanic steroid injection (5–22 dB). A randomized clinical trial, comparing the efficacy of topical IGF-1 treatment with that of intra-tympanic steroids for SSHL that is refractory to systemic steroids, is now underway, involving about 120 patients from all over Japan (University Hospital Medical Information Network Clinical Trials Registry under trial registration number UMIN000004366).

## CONCLUSION

In conclusion, IGF-1 is a promising medication for SNHL. IGF-1 activates both its downstream signaling pathways, the MEK/ERK and PI3K/Akt pathways in the cochlea, which causes more efficient effects on HC protection than activation of a single signal cascade. IGF-1 protects HCs from various injuries to the inner ear, including noise exposure, ischemia, and aminoglycoside treatment. A clinical trial of IGF-1 treatment on human SSHL as well as *in vivo* animal experiments has confirmed its efficacy against HC injuries.

## Conflict of Interest Statement

The authors declare that the research was conducted in the absence of any commercial or financial relationships that could be construed as a potential conflict of interest.

## References

[B1] Aburto M. R. Magarinos M. Leon Y. Varela-Nieto I. Sanchez-Calderon H. (2012). AKT signaling mediates IGF-I survival actions on otic neural progenitors. *PLoS ONE* 7:e30790 10.1371/journal.pone.0030790 PMC3264639 22292041

[B2] Adler H. J. Raphael Y. (1996). New hair cells arise from supporting cell conversion in the acoustically damaged chick inner ear. *Neurosci. Lett.* 205 17–20 10.1016/0304-3940(96)12367-3 8867010

[B3] Angunsri N. Taura A. Nakagawa T. Hayashi Y. Kitajiri S. Omi E. (2011). Insulin-like growth factor 1 protects vestibular hair cells from aminoglycosides. *Neuroreport* 22 38–43 10.1097/WNR.0b013e32834273e9 21127443

[B4] Armelin H. A. (1973). Pituitary extracts and steroid hormones in the control of 3T3 cell growth. *Proc. Natl. Acad. Sci. U.S.A.* 70 2702–2706 10.1073/pnas.70.9.2702 4354860 PMC427087

[B5] Attias J. Zarchi O. Nageris B. I. Laron Z. (2012). Cochlear hearing loss in patients with Laron syndrome. *Eur. Arch. Otorhinolaryngol.* 269 461–466 10.1007/s00405-011-1668-x 21735352

[B6] Baird R. A. Steyger P. S. Schuff N. R. (1996). Mitotic and nonmitotic hair cell regeneration in the bullfrog vestibular otolith organs. *Ann. N. Y. Acad. Sci.* 781 59–70 10.1111/j.1749-6632.1996.tb15693.x 8694449

[B7] Barrenas M. Landin-Wilhelmsen K. Hanson C. (2000). Ear and hearing in relation to genotype and growth in Turner syndrome. *Hear. Res.* 144 21–28 10.1016/S0378-5955(00)00040-X 10831862

[B8] Battaglia A. Pak K. Brors D. Bodmer D. Frangos J. A. Ryan A. F. (2003). Involvement of ras activation in toxic hair cell damage of the mammalian cochlea. *Neuroscience* 122 1025–1035 10.1016/j.neuroscience.2003.08.041 14643769

[B9] Bell T. J. Oberholtzer J. C. (2010). cAMP-induced auditory supporting cell proliferation is mediated by ERK MAPK signaling pathway. *J. Assoc. Res. Otolaryngol.* 11 173–185 10.1007/s10162-009-0205-8 20107853 PMC2862916

[B10] Blakesley V. A. Scrimgeour A. Esposito D. Le Roith D. (1996). Signaling via the insulin-like growth factor-I receptor: does it differ from insulin receptor signaling? *Cytokine Growth Factor Rev.* 7 153–159 10.1016/1359-6101(96)00015-9 8899293

[B11] Bonapace G. Concolino D. Formicola S. Strisciuglio P. (2003). A novel mutation in a patient with insulin-like growth factor 1 (IGF1) deficiency. *J. Med. Genet.* 40 913–917 10.1136/jmg.40.12.913 14684690 PMC1735341

[B12] Byl F. M. Jr. (1984). Sudden hearing loss: eight years’ experience and suggested prognostic table. *Laryngoscope* 94 647–661 10.1288/00005537-198405000-00014 6325838

[B13] Camarero G. Avendano C. Fernandez-Moreno C. Villar A. Contreras J. De Pablo F. (2001). Delayed inner ear maturation and neuronal loss in postnatal Igf-1-deficient mice. *J. Neurosci.* 21 7630–7641 11567053 10.1523/JNEUROSCI.21-19-07630.2001 PMC6762913

[B14] Camarero G. Villar M. A. Contreras J. Fernandez-Moreno C. Pichel J. G. Avendano C. (2002). Cochlear abnormalities in insulin-like growth factor-1 mouse mutants. *Hear. Res.* 170 2–11 10.1016/S0378-5955(02)00447-1 12208536

[B15] Cediel R. Riquelme R. Contreras J. Diaz A. Varela-Nieto I. (2006). Sensorineural hearing loss in insulin-like growth factor I-null mice: a new model of human deafness. *Eur. J. Neurosci.* 23 587–590 10.1111/j.1460-9568.2005.04584.x 16420467

[B16] Chan J. M. Stampfer M. J. Giovannucci E. Gann P. H. Ma J. Wilkinson P. (1998). Plasma insulin-like growth factor-I and prostate cancer risk: a prospective study. *Science* 279 563–566 10.1126/science.279.5350.563 9438850

[B17] Cohen S. (1962). Isolation of a mouse submaxillary gland protein accelerating incisor eruption and eyelid opening in the new-born animal. *J. Biol. Chem.* 237 1555–1562 13880319

[B18] Corwin J. T. Cotanche D. A. (1988). Regeneration of sensory hair cells after acoustic trauma. *Science* 240 1772–1774 10.1126/science.3381100 3381100

[B19] Finlay D. Healy V. Furlong F. O’Connell F. C. Keon N. K. Martin F. (2000). MAP kinase pathway signalling is essential for extracellular matrix determined mammary epithelial cell survival. *Cell Death Differ.* 7 302–313 10.1038/sj.cdd.4400652 10745275

[B20] Forge A. Li L. Corwin J. T. Nevill G. (1993). Ultrastructural evidence for hair cell regeneration in the mammalian inner ear. *Science* 259 1616–1619 10.1126/science.8456284 8456284

[B21] Froesch E. R. Buergi H. Ramseier E. B. Bally P. Labhart A. (1963). Antibody-suppressible and nonsuppressible insulin-like activities in human serum and their physiologic significance. An insulin assay with adipose tissue of increased precision and specificity. *J. Clin. Invest.* 42 1816–1834 10.1172/JCI104866 14083170 PMC289464

[B22] Fujiwara T. Hato N. Nakagawa T. Tabata Y. Yoshida T. Komobuchi H. (2008). Insulin-like growth factor 1 treatment via hydrogels rescues cochlear hair cells from ischemic injury. *Neuroreport* 19 1585–1588 10.1097/WNR.0b013e328311ca4b 18845939

[B23] Hakuba N. Gyo K. Yanagihara N. Mitani A. Kataoka K. (1997). Eﬄux of glutamate into the perilymph of the cochlea following transient ischemia in the gerbil. *Neurosci. Lett.* 230 69–71 10.1016/S0304-3940(97)00462-X 9259466

[B24] Hayashi Y. Yamamoto N. Nakagawa T. Ito J. (2013). Insulin-like growth factor 1 inhibits hair cell apoptosis and promotes the cell cycle of supporting cells by activating different downstream cascades after pharmacological hair cell injury in neonatal mice. *Mol. Cell. Neurosci.* 56 29–38 10.1016/j.mcn.2013.03.003 23511189

[B25] Hayashi Y. Yamamoto N. Nakagawa T. Ito J. (2014). Insulin-like growth factor 1 induces the transcription of Gap43 and Ntn1 during hair cell protection in the neonatal murine cochlea. *Neurosci. Lett.* 560 7–11 10.1016/j.neulet.2013.11.062 24333914

[B26] Hedgecock E. M. Culotti J. G. Hall D. H. (1990). The unc-5, unc-6, and unc-40 genes guide circumferential migrations of pioneer axons and mesodermal cells on the epidermis in C. elegans. *Neuron* 4 61–85 10.1016/0896-6273(90)90444-K 2310575

[B27] Holmström T. H. Tran S. E. Johnson V. L. Ahn N. G. Chow S. C. Eriksson J. E. (1999). Inhibition of mitogen-activated kinase signaling sensitizes HeLa cells to Fas receptor-mediated apoptosis. *Mol. Cell. Biol.* 19 5991–6002 10454546 10.1128/mcb.19.9.5991 PMC84476

[B28] Iwai K. Nakagawa T. Endo T. Matsuoka Y. Kita T. Kim T. S. (2006). Cochlear protection by local insulin-like growth factor-1 application using biodegradable hydrogel. *Laryngoscope* 116 529–533 10.1097/01.mlg.0000200791.77819.eb 16585854

[B29] Jayakumar C. Mohamed R. Ranganathan P. V. Ramesh G. (2011). Intracellular kinases mediate increased translation and secretion of netrin-1 from renal tubular epithelial cells. *PLoS ONE* 6:e26776 10.1371/journal.pone.0026776 PMC3202578 22046354

[B30] Jiang H. Sha S. H. Schacht J. (2006). Kanamycin alters cytoplasmic and nuclear phosphoinositide signaling in the organ of Corti in vivo. *J. Neurochem.* 99 269–276 10.1111/j.1471-4159.2006.04117.x 16903869

[B31] Johnson K. R. Marden C. C. Ward-Bailey P. Gagnon L. H. Bronson R. T. Donahue L. R. (2007). Congenital hypothyroidism, dwarfism, and hearing impairment caused by a missense mutation in the mouse dual oxidase 2 gene, Duox2. *Mol. Endocrinol.* 21 1593–1602 10.1210/me.2007-0085 17440044

[B32] Jones J. I. Clemmons D. R. (1995). Insulin-like growth factors and their binding proteins: biological actions. *Endocr. Rev.* 16 3–34 10.1210/edrv-16-1-3 7758431

[B33] Jorgensen J. M. Mathiesen C. (1988). The avian inner ear. Continuous production of hair cells in vestibular sensory organs, but not in the auditory papilla. *Naturwissenschaften* 75 319–320 10.1007/BF00367330 3205314

[B34] Kato H. Faria T. N. Stannard B. Roberts C. T. Jr. Leroith D. (1994). Essential role of tyrosine residues 1131, 1135, and 1136 of the insulin-like growth factor-I (IGF-I) receptor in IGF-I action. *Mol. Endocrinol.* 8 40–50 10.1210/mend.8.1.7512194 7512194

[B35] Koga K. Hakuba N. Watanabe F. Shudou M. Nakagawa T. Gyo K. (2003). Transient cochlear ischemia causes delayed cell death in the organ of Corti: an experimental study in gerbils. *J. Comp. Neurol.* 456 105–111 10.1002/cne.10479 12509868

[B36] Kopke R. D. Jackson R. L. Li G. Rasmussen M. D. Hoffer M. E. Frenz D. A. (2001). Growth factor treatment enhances vestibular hair cell renewal and results in improved vestibular function. *Proc. Natl. Acad. Sci. U.S.A.* 98 5886–5891 10.1073/pnas.101120898 11331776 PMC33308

[B37] Lambert P. R. (1994). Inner ear hair cell regeneration in a mammal: identification of a triggering factor. *Laryngoscope* 104 701–718 10.1288/00005537-199406000-00010 8196445

[B38] Langer R. Vacanti J. P. (1993). Tissue engineering. *Science* 260 920–926 10.1126/science.8493529 8493529

[B39] Laron Z. (1999). Somatomedin-1 (recombinant insulin-like growth factor-1): clinical pharmacology and potential treatment of endocrine and metabolic disorders. *BioDrugs* 11 55–70 10.2165/00063030-199911010-00006 18031115

[B40] Laviola L. Natalicchio A. Giorgino F. (2007). The IGF-I signaling pathway. *Curr. Pharm. Des.* 13 663–669 10.2174/138161207780249146 17346182

[B41] Lee K. Y. Nakagawa T. Okano T. Hori R. Ono K. Tabata Y. (2007). Novel therapy for hearing loss: delivery of insulin-like growth factor 1 to the cochlea using gelatin hydrogel. *Otol. Neurotol.* 28 976–981 10.1097/MAO.0b013e31811f40db 17704706

[B42] Lefebvre P. P. Malgrange B. Thiry M. Van De Water T. R. Moonen G. (2000). Epidermal growth factor upregulates production of supernumerary hair cells in neonatal rat organ of corti explants. *Acta Otolaryngol.* 120 142–145 10.1080/000164800750000784 11603759

[B43] Leon Y. Sanz C. Giraldez F. Varela-Nieto I. (1998). Induction of cell growth by insulin and insulin-like growth factor-I is associated with Jun expression in the otic vesicle. *J. Comp. Neurol.* 398 323–332 10.1002/(SICI)1096-9861(19980831)398:3<323::AID-CNE2>3.0.CO;2-1 9714146

[B44] Leon Y. Vazquez E. Sanz C. Vega J. A. Mato J. M. Giraldez F. (1995). Insulin-like growth factor-I regulates cell proliferation in the developing inner ear, activating glycosyl-phosphatidylinositol hydrolysis and Fos expression. *Endocrinology* 136 3494–3503 10.1210/endo.136.8.7628386 7628386

[B45] Lin F. R. Thorpe R. Gordon-Salant S. Ferrucci L. (2011). Hearing loss prevalence and risk factors among older adults in the United States. *J. Gerontol. A Biol. Sci. Med. Sci.* 66 582–590 10.1093/gerona/glr002 21357188 PMC3074958

[B46] Magarinos M. Aburto M. R. Sanchez-Calderon H. Munoz-Agudo C. Rapp U. R. Varela-Nieto I. (2010). RAF kinase activity regulates neuroepithelial cell proliferation and neuronal progenitor cell differentiation during early inner ear development. *PLoS ONE* 5:e14435 10.1371/journal.pone.0014435 PMC3010996 21203386

[B47] Malgrange B. Rigo J. M. Coucke P. Thiry M. Hans G. Nguyen L. (2002). Identification of factors that maintain mammalian outer hair cells in adult organ of Corti explants. *Hear. Res.* 170 48–58 10.1016/S0378-5955(02)00451-3 12208540

[B48] Marazita M. L. Ploughman L. M. Rawlings B. Remington E. Arnos K. S. Nance W. E. (1993). Genetic epidemiological studies of early-onset deafness in the U.S. school-age population. *Am. J. Med. Genet.* 46 486–491 10.1002/ajmg.1320460504 8322805

[B49] Murillo-Cuesta S. Camarero G. Gonzalez-Rodriguez A. De La Rosa L. R. Burks D. J. Avendano C. (2012). Insulin receptor substrate 2 (IRS2)-deficient mice show sensorineural hearing loss that is delayed by concomitant protein tyrosine phosphatase 1B (PTP1B) loss of function. *Mol. Med.* 18 260–269 10.2119/molmed.2011.00328 22160220 PMC3324951

[B50] Myers M. G. Jr. Sun X. J. Cheatham B. Jachna B. R. Glasheen E. M. (1993). IRS-1 is a common element in insulin and insulin-like growth factor-I signaling to the phosphatidylinositol 3’-kinase. *Endocrinology* 132 1421–1430 10.1210/endo.132.4.8384986 8384986

[B51] Nakagawa T. Ogino-Nishimura E. Hiraumi H. Sakamoto T. Yamamoto N. Ito J. (2012). Audiometric outcomes of topical IGF1 treatment for sudden deafness refractory to systemic steroids. *Otol. Neurotol.* 33 941–946 10.1097/MAO.0b013e31825f251a 22772021

[B52] Nakagawa T. Sakamoto T. Hiraumi H. Kikkawa Y. S. Yamamoto N. Hamaguchi K. (2010). Topical insulin-like growth factor 1 treatment using gelatin hydrogels for glucocorticoid-resistant sudden sensorineural hearing loss: a prospective clinical trial. *BMC Med.* 8:76 10.1186/1741-7015-8-76 PMC3000370 21108784

[B53] Oesterle E. C. Tsue T. T. Rubel E. W. (1997). Induction of cell proliferation in avian inner ear sensory epithelia by insulin-like growth factor-I and insulin. *J. Comp. Neurol.* 380 262–274 10.1002/(SICI)1096-9861(19970407)380:2<262::AID-CNE8>3.0.CO;2-1 9100136

[B54] Okano T. Xuan S. Kelley M. W. (2011). Insulin-like growth factor signaling regulates the timing of sensory cell differentiation in the mouse cochlea. *J. Neurosci.* 31 18104–18118 10.1523/JNEUROSCI.3619-11.2011 22159122 PMC6634146

[B55] Pagès G. Lenormand P. L’Allemain G. Chambard J. C. Meloche S. Pouysségur J. (1993). Mitogen-activated protein kinases p42mapk and p44mapk are required for fibroblast proliferation. *Proc. Natl. Acad. Sci. U.S.A.* 90 8319–8323 10.1073/pnas.90.18.8319 8397401 PMC47347

[B56] Park J. Y. Park Y. H. Shin D. H. Oh S. H. (2007). Insulin-like growth factor binding protein (IGFBP)-mediated hair cell survival on the mouse utricle exposed to neomycin: the roles of IGFBP-4 and IGFBP-5. *Acta Otolaryngol. Suppl.* 22–29 10.1080/03655230701624822 17882566

[B57] Rinderknecht E. Humbel R. E. (1976a). Amino-terminal sequences of two polypeptides from human serum with nonsuppressible insulin-like and cell-growth-promoting activities: evidence for structural homology with insulin B chain. *Proc. Natl. Acad. Sci. U.S.A.* 73 4379–4381 10.1073/pnas.73.12.4379 1069990 PMC431462

[B58] Rinderknecht E. Humbel R. E. (1976b). Polypeptides with nonsuppressible insulin-like and cell-growth promoting activities in human serum: isolation, chemical characterization, and some biological properties of forms I and II. *Proc. Natl. Acad. Sci. U.S.A.* 73 2365–2369 10.1073/pnas.73.7.2365 1065887 PMC430569

[B59] Rinderknecht E. Humbel R. E. (1978). The amino acid sequence of human insulin-like growth factor I and its structural homology with proinsulin. *J. Biol. Chem.* 253 2769–2776 632300

[B60] Riquelme R. Cediel R. Contreras J. La Rosa Lourdes R. D. Murillo-Cuesta S. Hernandez-Sanchez C. (2010). A comparative study of age-related hearing loss in wild type and insulin-like growth factor I deficient mice. *Front. Neuroanat.* 4:27 10.3389/fnana.2010.00027 PMC2907134 20661454

[B61] Ruben R. J. (1967). Development of the inner ear of the mouse: a radioautographic study of terminal mitoses. *Acta Otolaryngol. Suppl.* 220 221–244 6067797

[B62] Ryals B. M. Rubel E. W. (1988). Hair cell regeneration after acoustic trauma in adult *Coturnix* quail. *Science* 240 1774–1776 10.1126/science.3381101 3381101

[B63] Salmon W. D. Jr. Daughaday W. H. (1957). A hormonally controlled serum factor which stimulates sulfate incorporation by cartilage in vitro. *J. Lab. Clin. Med.* 49 825–836 13429201

[B64] Sanchez-Calderon H. Rodriguez-De La Rosa L. Milo M. Pichel J. G. Holley M. Varela-Nieto I. (2010). RNA microarray analysis in prenatal mouse cochlea reveals novel IGF-I target genes: implication of MEF2 and FOXM1 transcription factors. *PLoS ONE* 5:e8699 10.1371/journal.pone.0008699 PMC2810322 20111592

[B65] Sanz C. Leon Y. Troppmair J. Rapp U. R. Varela-Nieto I. (1999). Strict regulation of c-Raf kinase levels is required for early organogenesis of the vertebrate inner ear. *Oncogene* 18 429–437 10.1038/sj.onc.1202312 9927199

[B66] Selivanova O. Brieger J. Heinrich U. R. Mann W. (2007). Akt and c-Jun N-terminal kinase are regulated in response to moderate noise exposure in the cochlea of guinea pigs. *ORL J. Otorhinolaryngol. Relat. Spec.* 69 277–282 10.1159/000103871 17565230

[B67] Shepherd P. R. Withers D. J. Siddle K. (1998). Phosphoinositide 3-kinase: the key switch mechanism in insulin signalling. *Biochem. J.* 333(Pt 3) 471–490 9677303 10.1042/bj3330471 PMC1219607

[B68] Vanhaesebroeck B. Alessi D. R. (2000). The PI3K-PDK1 connection: more than just a road to PKB. *Biochem. J.* 346 561–576 10.1042/0264-6021:3460561 10698680 PMC1220886

[B69] Walenkamp M. J. Karperien M. Pereira A. M. Hilhorst-Hofstee Y. Van Doorn J. Chen J. W. (2005). Homozygous and heterozygous expression of a novel insulin-like growth factor-I mutation. *J. Clin. Endocrinol. Metab.* 90 2855–2864 10.1210/jc.2004-1254 15769976

[B70] Wang W. Reeves W. B. Pays L. Mehlen P. Ramesh G. (2009a). Netrin-1 overexpression protects kidney from ischemia reperfusion injury by suppressing apoptosis. *Am. J. Pathol.* 175 1010–1018 10.2353/ajpath.2009.090224 19700747 PMC2731120

[B71] Wang W. Reeves W. B. Ramesh G. (2009b). Netrin-1 increases proliferation and migration of renal proximal tubular epithelial cells via the UNC5B receptor. *Am. J. Physiol. Renal Physiol.* 296 F723–F729 10.1152/ajprenal.90686.2008 19211685

[B72] Wang Y. Z. Wong Y. C. (1998). Sex hormone-induced prostatic carcinogenesis in the noble rat: the role of insulin-like growth factor-I (IGF-I) and vascular endothelial growth factor (VEGF) in the development of prostate cancer. *Prostate* 35 165–177 10.1002/(SICI)1097-0045(19980515)35:3<165::AID-PROS2>3.0.CO;2-G 9582085

[B73] Warchol M. E. Lambert P. R. Goldstein B. J. Forge A. Corwin J. T. (1993). Regenerative proliferation in inner ear sensory epithelia from adult guinea pigs and humans. *Science* 259 1619–1622 10.1126/science.8456285 8456285

[B74] Werner H. Leroith D. (2014). Insulin and insulin-like growth factor receptors in the brain: physiological and pathological aspects. *Eur. Neuropsychopharmacol.* 10.1016/j.euroneuro.2014.01.020 [Epub ahead of print] 24529663

[B75] White M. F. (1997). The insulin signalling system and the IRS proteins. *Diabetologia* 40(Suppl. 2), S2–S17 10.1007/s001250051387 9248696

[B76] Wolf E. Jehle P. M. Weber M. M. Sauerwein H. Daxenberger A. Breier B. H. (1997). Human insulin-like growth factor I (IGF-I) produced in the mammary glands of transgenic rabbits: yield, receptor binding, mitogenic activity, and effects on IGF-binding proteins. *Endocrinology* 138 307–313 10.1210/endo.138.1.4877 8977418

[B77] Woods K. A. Camacho-Hubner C. Savage M. O. Clark A. J. (1996). Intrauterine growth retardation and postnatal growth failure associated with deletion of the insulin-like growth factor I gene. *N. Engl. J. Med.* 335 1363–1367 10.1056/NEJM199610313351805 8857020

[B78] Yamashita H. Oesterle E. C. (1995). Induction of cell proliferation in mammalian inner-ear sensory epithelia by transforming growth factor alpha and epidermal growth factor. *Proc. Natl. Acad. Sci. U.S.A.* 92 3152–3155 10.1073/pnas.92.8.3152 7724532 PMC42123

[B79] Yamauchi K. Pessin J. E. (1994). Insulin receptor substrate-1 (IRS1) and Shc compete for a limited pool of Grb2 in mediating insulin downstream signaling. *J. Biol. Chem.* 269 31107–31114 7983051

[B80] Young S. Wong M. Tabata Y. Mikos A. G. (2005). Gelatin as a delivery vehicle for the controlled release of bioactive molecules. *J. Control. Release* 109 256–274 10.1016/j.jconrel.2005.09.023 16266768

[B81] Zheng J. L. Helbig C. Gao W. Q. (1997). Induction of cell proliferation by fibroblast and insulin-like growth factors in pure rat inner ear epithelial cell cultures. *J. Neurosci.* 17 216–226 8987750 10.1523/JNEUROSCI.17-01-00216.1997 PMC6793686

